# Functional methods for evaluating the efficacy of retinal optogenetic therapy for vision restoration

**DOI:** 10.3389/fnins.2026.1812539

**Published:** 2026-04-23

**Authors:** Grace A. Borchert, Hoda Shamsnajafabadi, Kanmin Xue, Robert E. MacLaren, Samantha R. De Silva, Jasmina Cehajic-Kapetanovic

**Affiliations:** 1 Nuffield Laboratory of Ophthalmology, Nuffield Department of Clinical Neurosciences, University of Oxford, Oxford, United Kingdom; 2 Oxford Eye Hospital, Oxford University Hospitals NHS Foundation Trust, Oxford, United Kingdom

**Keywords:** clinical trials, functional assay, optogenetics, preclinical, vision restoration

## Abstract

Optogenetic therapy is a promising strategy for vision restoration. Functional assays have an important role in assessing the modulation of neural activity in response to light stimulation. Each functional assay needs to be carefully considered and evaluated for the preclinical development of optogenetic approaches to work toward meaningful vision restoration. Each strategy contributes to understanding the efficacy of vision restoration and the physiology of retinal optogenetic therapy. At a molecular level, bioluminescence resonance energy-transfer based and G protein coupling assays can be used. Calcium imaging provides measurements with useful spatial and temporal resolution using fluorescent calcium indicators at the cellular level. Electrophysiological tests can include *ex vivo* recordings by patch-clamping at single-cell resolution, multielectrode array recordings at the network level, and *in vivo* recordings at the lateral geniculate nucleus and cortical levels. Behavioural tests such as light avoidance, optomotor response and visual discrimination assess functional restoration *in vivo.* In this review, each functional assay is discussed in the context of retinal optogenetic therapy with notable examples that have demonstrated vision restoration. The advantages, disadvantages, and limitations of each assay are critically compared to highlight their relative scientific value and applicability across different stages of development. This provides insight into how these methods can be integrated within a translational framework, from molecular validation to behavioural outcomes, to better inform the design of preclinical studies. As clinical trials in optogenetic therapy continue to expand, improved alignment between preclinical functional assays and clinically meaningful endpoints will be essential to maximise translational success.

## Introduction

Optogenetics is a method of neuromodulation that uses light to activate cells by the ectopic expression of light-sensitive opsins (McClements et al., 2020). Since the conversion of light to electrical signals occurs in the eye via phototransduction in rods and cones, a significant application of optogenetics is to induce light sensitivity in the remaining cells and architecturally intact retina in end-stage retinal degeneration to restore vision (Cehajic-Kapetanovic et al., 2015; De Silva et al., 2017). There are many applications of optogenetic therapy, regardless of the underlying cause of retinal degeneration. For example, inherited retinal diseases (IRDs), the most common cause of visual impairment in the working age, and advanced age-related macular degeneration, the leading cause of blindness in people over the age of 50 years in the developed world. Optogenetic therapy holds promising potential in vision restoration where there has not been treatment available before and this is regardless of the underlying cause. Several photosensitive proteins have been evaluated as optogenetic tools, such as microbial (e.g., ChR2, ChrimsonR, ChronosFP) and human opsins (e.g., melanopsin, rhodopsin, cone opsins), as well as chimeras and engineered proteins, that are either G protein coupled receptors or ion channels. Each differs in sensitivity, peak activation wavelength, kinetics and immunogenicity, which need to be carefully considered for future clinical translation. To measure each optogenetic tool, functional assays provide a way to evaluate changes at the cellular, tissue, or behavioural level to determine potential real-world vision restoration.

Functional assays in optogenetics have an important role in confirming neuronal modulation in response to light, assessing the efficacy and guiding optimisation in therapeutic development for vision restoration. However, there is a current lack of critical comparison between functional assays to assess the efficacy of emerging novel optogenetic treatments. Different functional assays provide evidence at the cellular (e.g., patch-clamp, Bioluminescence Resonance Energy Transfer (BRET) assay), network (e.g., multielectrode array (MEA), calcium imaging), and behavioural levels following optogenetic light stimulation. This is useful for understanding neuronal circuits in normal physiology, investigating a wide range of diseases, and evaluating optogenetic therapeutic strategies (Ballister et al., 2018). Calcium imaging allows visualisation of light-evoked activity; meanwhile, patch clamp provides a precise, high-resolution view at the cellular level. MEA captures extracellular activity from a neuronal network simultaneously, and the BRET assay assesses intracellular signalling changes after opsin interaction. Functional electrodiagnostic tests, such as full-field electroretinography (ERG) and visual evoked potentials (VEP), measure *in vivo* overall retinal function and cortical responses, respectively. Visually evoked behavioural assays are tests used to assess visual function in animal models by measuring how animals behave in response to visual stimuli. These assays are crucial tools in evaluating the effectiveness of optogenetic treatments in models of retinal degeneration. They provide functional evidence of restored vision, especially when objective electrophysiological measures (e.g., ERG or VEP) show ambiguous results. Behavioural outcomes also relate more directly to perceived vision, helping to translate findings from preclinical models to potential human experiences.

While there have been significant advances in the field, optogenetic functional assays each have their advantages and disadvantages in evaluating the efficacy and safety. The aim of this review is to discuss the functional assays of optogenetics, their applications, and to compare their advantages, disadvantages, and limitations for evaluating vision restoration. Despite the breadth of available methods, there remains a lack of standardisation and a clear translational framework to guide how these assays should be selected, interpreted, and integrated across different stages of development. This has contributed to variability in reported outcomes and challenges in comparing findings across studies, ultimately limiting the ability to identify best-in-class optogenetic approaches. As the field advances toward late-stage clinical trials, the selection of appropriate functional endpoints has emerged as a key determinant of translational success. This review therefore aims to provide a structured and critical appraisal of current functional assays, placing them within a coherent pipeline from molecular to behavioural assessment, and highlighting how they can be prioritised and aligned with clinically meaningful measures of vision restoration.

## G-protein coupling assays for G-protein coupled based optogenetic tools

Optogenetic therapy can modulate stimulatory G protein (Gs), inhibitory G protein (Gi), other G protein (Go) and chimeric G protein (GsX) and real-time biosensor methods are needed quantify G-protein coupled receptor (GPCR) protein coupling and downstream activation. Quantifying these early interactions provide a direct read out of optogenetic function over time. Engineered G-protein subunits are fused to fluorescent or luminescent tags that report changes in G-protein dissociation after stimulation. A GPCR actuator, such as an optogenetic GPCR, is expressed with a luminescent donor on one G-protein component and a fluorescent acceptor on another. At baseline, the donor and acceptor are in close proximity, causing a high signal. Upon activation, the receptor leads to GDP-GTP exchange or dissociation of the G protein subunits. This allows for a quantification of real-time magnitude and kinetics of the signalling pathway. A live cell assay of GPCR coupling allows for screening of optogenetic tools that modulate Go and Gi signalling using GsX assays (Ballister et al., 2018).

**Figure 1:**
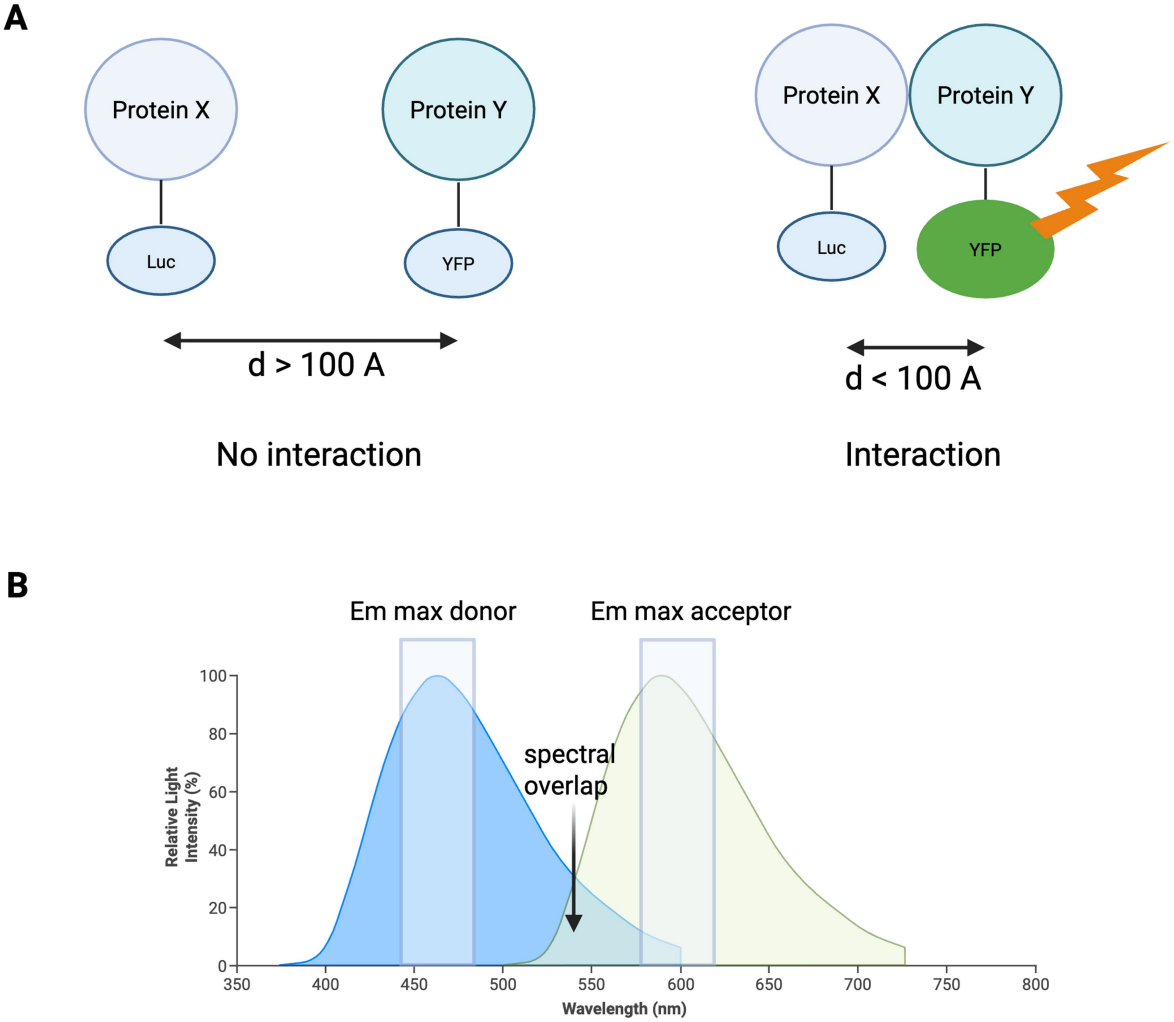
Principles of the bioluminescence resonance energy transfer (BRET) assay. Two proteins of interest, X and Y, have been fused to either a donor luciferase (Luc, maximal emission 475 nm) or an acceptor fluorescent protein (YFP, maximal emission 530 nm), respectively. **(A)** No interaction between protein X and Y, so there is no transfer of energy between Luc and YFP. Interaction between proteins X and Y results in energy transfer from Luc to YFP, increasing the light emitted by YFP. **(B)** Properties of donor and acceptor molecules for BRET, with the donor overlapping the excitation spectrum of the acceptor.

G-protein activation assays, particularly based on BRET, are relevant for GPCR based opsins (Rodgers et al., 2023). The BRET assay has been validated for opsins and their signalling (dissociation of G-protein heterotrimer) to provide a measure of changes in opsin state. Energy is transferred from a ‘donor’ luciferase enzyme to an ‘acceptor’ fluorescent protein (Xie et al., 2011). The most common donor is *Renilla* luciferase, and the acceptor is Yellow Fluorescent protein (YFP) (Deriziotis et al., 2014). The efficiency of energy transfer is determined by the distance between the donor and acceptor proteins, which are either fused with luciferase or YFP. The distance range where energy can be transferred is at a conventional protein dimension (<100 Ångströms, A) which provides a sensitive method to detect weak, transient or dependent biochemical protein interactions in a cell (Dolino et al., 2014) (Figure 1).

The BRET assay has useful applications in quantifying the functional activity of optogenetic tools through their downstream interactions, for instance, how a receptor activates with G-proteins (Masuho et al., 2015). The main advantage is the quantification of temporal resolution and kinetics, which can be evaluated with real-time monitoring of the dynamic processes of protein interactions. The disadvantages of the BRET assay are that it can be technically difficult to set up and there needs to be an appropriate selection of donor and acceptor molecules to control expression levels. There is potential for spectral overlap and challenges with signal stability depending on the timing of the protein–protein interactions. The intensity of the BRET assay signal is determined by several factors. The distance between the BRET partners is inversely proportional to transfer signal intensity, where the BRET signal reduces when the distance between the acceptor and donor increases (El Khamlichi et al., 2019). When the distance decreases, there is an increased overlap of the emission spectrum of the donor and the excitation spectrum of the acceptor (Brown et al., 2015). The orientation of the BRET assay donor and acceptors also affects the dipole–dipole nature of resonance energy transfer (Xu et al., 1999). The ratio between the donor and acceptor also affects the intensity of the BRET assay signal due to saturation, where the signal increases in a hyperbolic nature until it reaches a plateau where all donors have been in proximity of acceptor molecules (El Khamlichi et al., 2019).

While G-protein coupling assays such as BRET provide a sensitive and quantitative readout of early GPCR signalling, they remain a reductionist measure of optogenetic function. These assays primarily capture proximal molecular events, such as G-protein activation and dissociation, but do not directly reflect downstream neuronal excitability, network integration, or perceptual visual outcomes. This distinction is particularly relevant for optogenetic therapies, where the ultimate goal is restoration of meaningful vision rather than receptor-level activation alone. As such, strong coupling efficiency *in vitro* does not necessarily translate into functional efficacy in retinal circuits *in vivo*. Compared to electrophysiological approaches such as patch-clamp or multi-electrode array recordings, G-protein assays lack spatial and functional context, and therefore should be considered as early-stage screening tools rather than definitive measures of therapeutic performance.

## Calcium imaging

**Figure 2:**
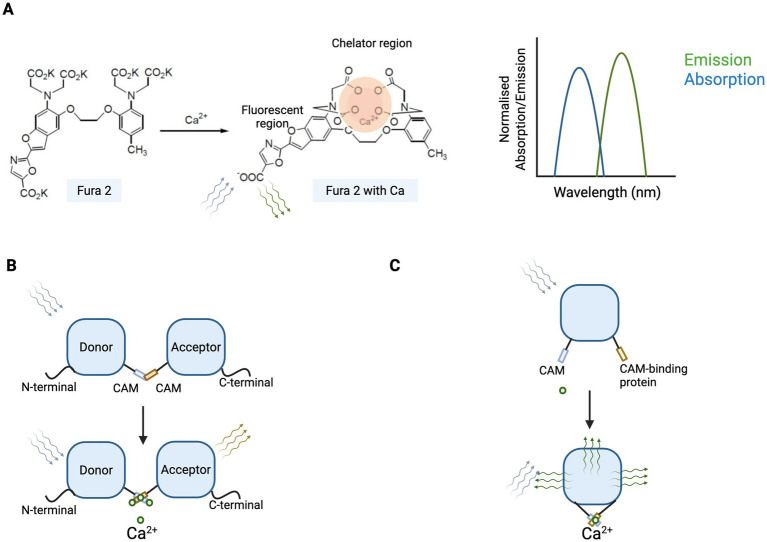
Types of calcium indicators, including chemical and genetically encoded variants. Chemical calcium indicator binding to calcium ion leads to fluorescence **(A)**. Genetically encoded calcium indicators (GECI) based on fluorescence resonance energy transfer (FRET) **(B)** and single fluorophore GECI resulting in a conformational change and fluorescence **(C)**. CAM = calmodulin termini.

Functional imaging of calcium is an important measure of neuronal activity as a result of optogenetic activation (de Melo Reis et al., 2020) and allows visualisation and quantification of neuronal activity measured directly by the detection of changes in intracellular calcium. Calcium imaging uses calcium indicators, which increase in fluorescence following the binding of calcium ions (Ca^2+^). Calcium Ca^2+^ signals can lead to three types of response: transient (brief influx through the membrane channels), sustained (sustained high calcium from internal and external stores) or oscillatory (successive brief increases in the free intracellular calcium) (Bagur and Hajnóczky, 2017). There are two forms of calcium indicators: chemical indicators and genetically encoded calcium indicators (Figure 2). Chemical indicators involve small molecules which chelate calcium ions. Each is generated based on an ethylene glycerol-bis-tetraacetic acid (EGTA) homologue, 1,2-bis(2-aminophenoxy)ethane-tetraacetic acid (BAPTA), which has a selectivity for calcium ions (Ca^2+^). The chemical calcium indicators include fluorescent dyes such as fura-2, fluo-3, fluo-4, indo-1, and calcium green-1 (Paredes et al., 2008). When the indicator enters the cell, esterases free the carboxyl group and the indicator is then able to bind to calcium. The binding of a Ca^2+^ ion to a fluorescent indicator molecule result in fluorescence, emission or excitation wavelength shift. Alternatively, genetically encoded calcium indicators (GECI) are useful tools which do not need to be acutely loaded into cells, but rather the genes encoding these proteins can be introduced by transfection (Oh et al., 2019; Weitz et al., 2013). While some GECI report calcium by direct emission of photons, others depend on fluorescent protein reporters such as GFP, eGFP and YFP.

Calcium imaging is a sensitive measure for monitoring the dynamics of calcium during neuronal activity (Cameron et al., 2017). In optogenetics, it is a powerful functional assay to evaluate light responsiveness in cells expressing ectopic optogenetic tools, and plays a critical role in the preclinical screening pipeline for retinal optogenetic therapies. By measuring light-evoked intracellular calcium influxes (evaluating the change in the fluorescence of the indicator dye as calcium enters the cell), this method allows researchers to determine whether cells expressing candidate opsins are functionally light-sensitive. Calcium imaging can be applied *in vitro* to systems such as HEK293 cells, neuronal cell lines such as Neuro2A cells, or cultured hippocampal pyramidal cells, enabling high-throughput evaluation of multiple opsin variants. After transient transfection or AAV vector transduction of cells with opsins, imaging can be performed, for example, using a Fluo-4 calcium indicator, at room temperature. The cells are exposed to intermittent light impulses that stimulate the opsin, leading to changes in calcium currents and release of fluorescence from the calcium dye for imaging. This approach is particularly well-suited for microbial opsins like ChR2 (Lin et al., 2009) and its enhanced derivatives (e.g., CatCh, ChrimsonR, or ChRmine), which directly mediate ion influx and trigger depolarisation-driven calcium entry upon light stimulation. For example, Fura-2 calcium imaging was performed on CatCh-expressing HEK293 cells to quantify the increase in Ca^2+^ permeability of CatCh compared to ChR (Kleinlogel et al., 2011). Calcium imaging is also suitable for GPCR-based human opsins, including melanopsin, rhodopsin, and engineered chimeric proteins (e.g., rhodopsin fused with arrestin or G-protein signalling domains), which activate intracellular signalling cascades leading to calcium mobilisation.

This approach was adapted using aequorin, a calcium-sensitive photoprotein that emits bioluminescence in response to intracellular calcium changes, to enable the quantification of light-induced calcium signalling in transfected HEK293 cells (Bailes and Lucas, 2013). The study focused on characterizing human melanopsin, a non-visual opsin expressed in intrinsically photosensitive retinal ganglion cells (ipRGCs). They showed that melanopsin forms a blue light-sensitive pigment (*λ* max ≈ 479 nm) and triggers large, sustained increases in intracellular calcium via Gq/11-coupled signalling, making it highly detectable with calcium imaging methods. In contrast, cells expressing rhodopsin in the same experimental system showed minimal calcium elevation, consistent with its canonical coupling to Gi/o-type pathways that inhibit adenylyl cyclase rather than elevate intracellular calcium. This difference underscores the pathway-specific limitations of calcium-based assays—while highly effective for opsins like melanopsin that activate calcium-elevating cascades, such assays may underrepresent the functional activity of opsins like rhodopsin. A better predictor of vertebrate opsin efficacy (and their chimeras and mutant variants) may therefore be their ability to couple to G proteins. Importantly, calcium imaging can distinguish differences in kinetics, light sensitivity, and signalling pathways among various opsins, allowing identification of top-performing candidates based on physiologically relevant response profiles. These insights are essential for prioritising constructs with optimal properties for *in vivo* validation in animal models and eventual translation to human trials.

In addition to the single-cell level, calcium imaging can be used at a population level, providing high spatial resolution of the neuronal network activity. The non-invasive nature of the approach makes it also suitable for *in vivo* assays (Russell, 2011; Stosiek et al., 2003). This has been used with calcium-AM dyes and retinal wholemounts for over 24 h after loading, and evoked calcium responses (Cameron et al., 2016; Singh et al., 2013). However, applying calcium imaging to retinal wholemounts is limited by the use of excitation light to activate the opsin which can also stimulate photoreceptors or the calcium indicator, confounding interpretation, the thickness of the tissue and light scattering.

The limitations are that temporally, calcium is slower than membrane potentials, which means that it cannot be measured as precisely as electrophysiology outputs; the intensity quantitatively does not equate directly to calcium concentrations or action potentials; there can be photobleaching and phototoxicity and signal overlap with dense neural tissue.

To overcome photobleaching, two-photon calcium imaging and *in vivo* adaptive optics scanning light ophthalmoscopy (AOSLO) assays have been used for functional testing of optogenetic therapies. Two-photon calcium imaging uses infrared excitation which decreases spectral overlap with optogenetic actuators and allows deeper characterisation of retinal circuits. Complementing *ex vivo* approaches, AOSLO provides a more powerful tool for cellular analysis of retinal activity in retinal degeneration mouse models, such as *rd10* mice, and in non-human primates. By stabilising ocular motion and using low-energy light, AOSLO enables direct quantification of optogenetic functional vision restoration.

Optogenetic therapy has restored retinal activity, as measured by fluorescence AOSLO and calcium imaging (McGregor et al., 2020). Co-expression of the fluorescently labelled optogenetic ChrimsonR and the calcium indicator GCaMPs was achieved by intravitreal co-injection of AAV2. CAG.ChrimsonR:tdTomato (dose 1.05E+12) and AAV2.CAG.GCaMP6s (dose 1.9E+13) in a non-human primate retina from 2 weeks after injection. Retinal ganglion cells responded to optogenetic stimulation when recorded by calcium imaging AOSLO at 3 months. Optogenetic responses in retinal ganglion cells in primate remained for at least 1 year following photoreceptor degeneration with a comparable sensitivity index to optogenetic responses in intact retina. This provides a promising proof-of-concept for vision restoration that depends on the reactivation of preserved retinal ganglion cells. However, a limitation of this functional assay is the long-time constant of the calcium indicator, GCaMP6s, at 0.6 s, which restricts the evaluation of the temporal resolution of the visual restoration at oscillations above 1 Hz (McGregor et al., 2020).

Overall, calcium imaging gives a sensitive and scalable readout of optogenetic activation. Nonetheless, it remains an indirect surrogate of neuronal function and needs to be interpreted with caution in the context of translational efficacy. Calcium signals reflect intracellular second messenger dynamics rather than direct membrane depolarisation, and therefore may overestimate functional responsiveness, particularly in systems where calcium mobilisation is amplified downstream of GPCR signalling. This is especially relevant when comparing microbial opsins, which induce direct ion flux, with vertebrate or chimeric opsins that rely on intracellular signalling cascades. Calcium imaging may hence favour opsins that couple strongly to calcium pathways while underrepresenting those operating through alternative signalling mechanisms. In addition, compared to electrophysiological approaches such as patch-clamp or MEA recordings, calcium imaging lacks precise temporal resolution and does not fully capture spiking activity or network integration, both of which are critical for meaningful visual restoration. Calcium imaging thus remains a highly valuable tool for high-throughput screening and initial functional validation, while downstream electrophysiological and behavioural assays are needed to more accurately predict *in vivo* performance and translational potential of optogenetic therapies.

## Patch clamp

Patch clamp provides a technique to investigate ionic currents *in vitro* with isolated cells or *ex vivo* with tissue structures (Hill and Stephens, 2021). The advantage of *in vitro* isolated cell patch clamp is precision and a controlled environment, while *ex vivo* allows for the study of neuronal circuits with an *in vivo* environment and 3-dimensional structure. Patch clamp can be performed using a voltage clamp, where the voltage across the membrane is controlled, and the currents are recorded. Alternatively, it can be conducted using a current clamp, in which the current across the membrane is controlled, and the voltage is recorded. The technique was developed by Erwin Neher and Bert Sakmann, and it received the Nobel Prize in Physiology or Medicine in 1991 (Vyklický, 1992).

In practice, the setup involves a micropipette, a hollow glass tube, and a recording electrode connected to an amplifier in contact with the membrane. A ground electrode is then submerged in a bath, containing ions to mimic the physiological environment, surrounding the cell or tissue. A current is then formed between the recording and ground electrode. This is then recorded and analysed using a computer interface.

**Figure 3:**
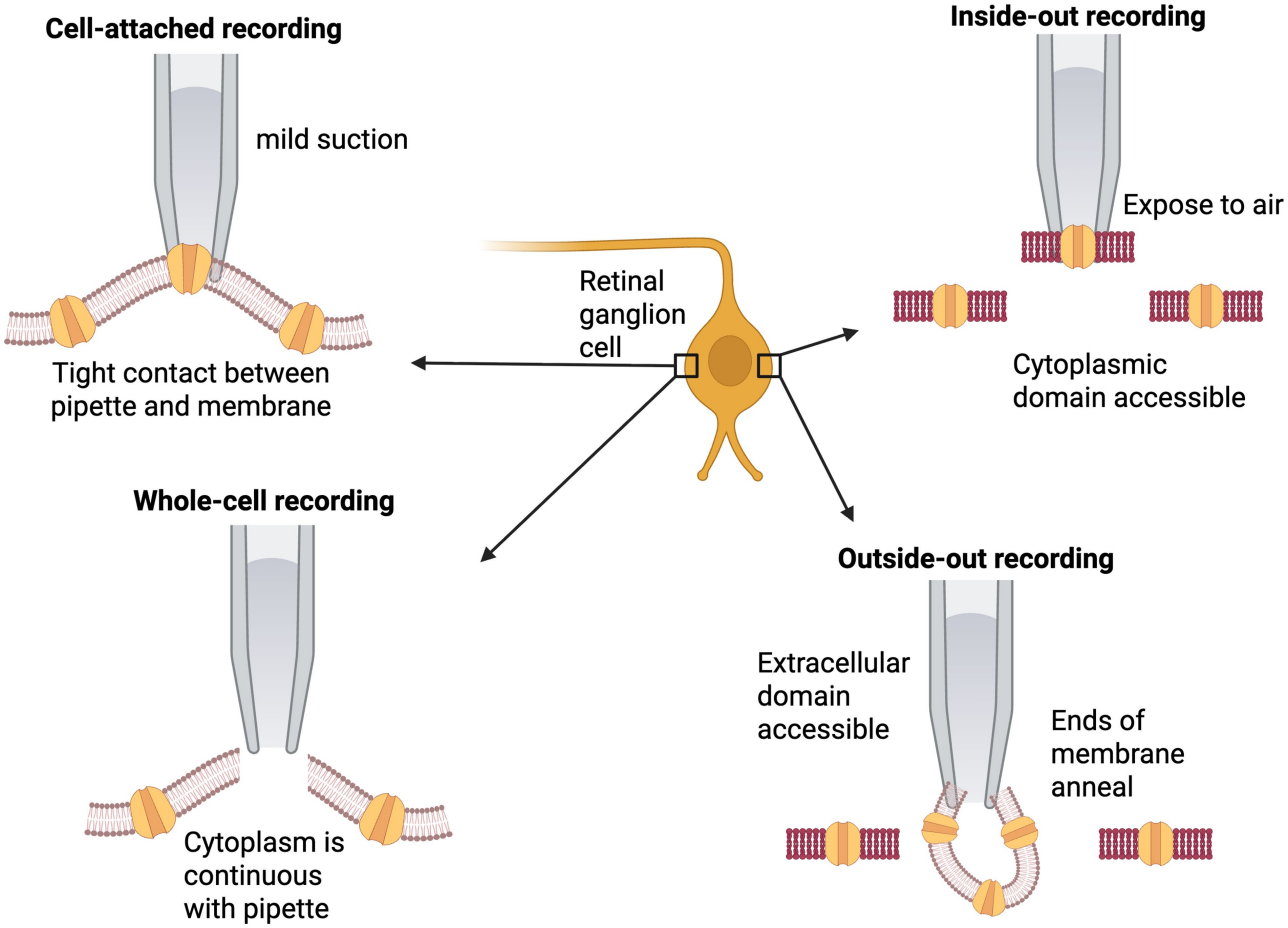
Methods of patch clamp: Cell-attached where the pipette is in contact with the membrane, whole-cell where there is a brief strong suction to attain access to the cytoplasm, inside-out retracts a patch of membrane so the cytoplasmic surface is exposed, and outside-out method with the retraction of a patch that forms a small vesicle structure with the cytoplasmic surface facing outwards.

There are several patch-clamp techniques that can be used depending on the primary research question (Figure 3) (Ghovanloo et al., 2024). The outside-in and inside-out techniques are considered “excised” patch techniques since the membrane is excised from the cell. Whole-cell patch clamping allows for investigating the electrical activity of the cell, rather than single channel currents. Cell-attached patch clamping is particularly useful for studying ion channel activity in a small patch of the membrane. It involves a pipette that is sealed to the cell membrane, the intracellular contents remains unchanged and allows for high-resolution recordings of individual ion channel activity. Although it is limited by background currents from nearby cells, it is not possible to control the intracellular environment. Whole-cell mode is where the membrane patch is disrupted to allow for access to the internal cell for recording across the whole cell membrane. It is comprehensive, and the intracellular solution can be controlled, although unstable, and there can be a loss of cellular components in the process. Meanwhile, the inside-out mode detaches a patch of membrane to expose the intracellular aspect to the external bath. There is direct intracellular access, which is useful for studying intracellular molecules and their downstream activity. In contrast, outside-out mode creates a vesicle-like structure with the extracellular surface in contact with the bath solution. The outside-out mode is helpful in assessing the ligand-gated ion channel stability and allows for a direct study of isolated channel properties (removal of ligands, neurotransmitters and pharmacological agents) following optogenetic therapy testing while maintaining electrical access to the intracellular side.

Patch clamp plays a fundamental role in assessing preclinical optogenetic therapies for vision restoration. For example, bReaChES expression in retinal ganglion cells were able to restore vision measured by single-cell patch-clamp recordings in *rd1* mice retinal ganglion cells (Too et al., 2022). Meanwhile, melanopsin-mGluR6 chimera targeting bipolar cells showed vision restoration and patch clamp was used to assess the function of retinal ganglion cells in *rd1* mice (Kralik et al., 2022). Patch clamp has the advantage of directly measuring functional response with a high temporal and spatial resolution. However, there are several disadvantages in being a technically challenging setup and performance, invasive, and cellular level measurements (Yajuan et al., 2012).

Although rightly considered the gold-standard technique for measuring cellular electrophysiological responses, patch clamp remains inherently limited when considered in the context of vision restoration. It measures precise quantification of ionic currents and membrane potentials, which are limited to single cells or small populations. In addition, recordings are often performed under highly controlled ex vivo conditions, which may not fully replicate the complex physiological environment of the degenerating retina. Compared to calcium imaging, patch clamp offers superior temporal resolution but lacks scalability, making it less suitable for high-throughput screening. Similarly, while it provides more direct functional neuronal readouts compared to molecular assays such as G-protein coupling, it is still unable to capture how optogenetic signals are integrated across retinal circuits or propagated to higher visual centres. It hence needs to be integrated with network-level and behavioural assessments to strengthen translational relevance.

## Multielectrode array

**Figure 4:**
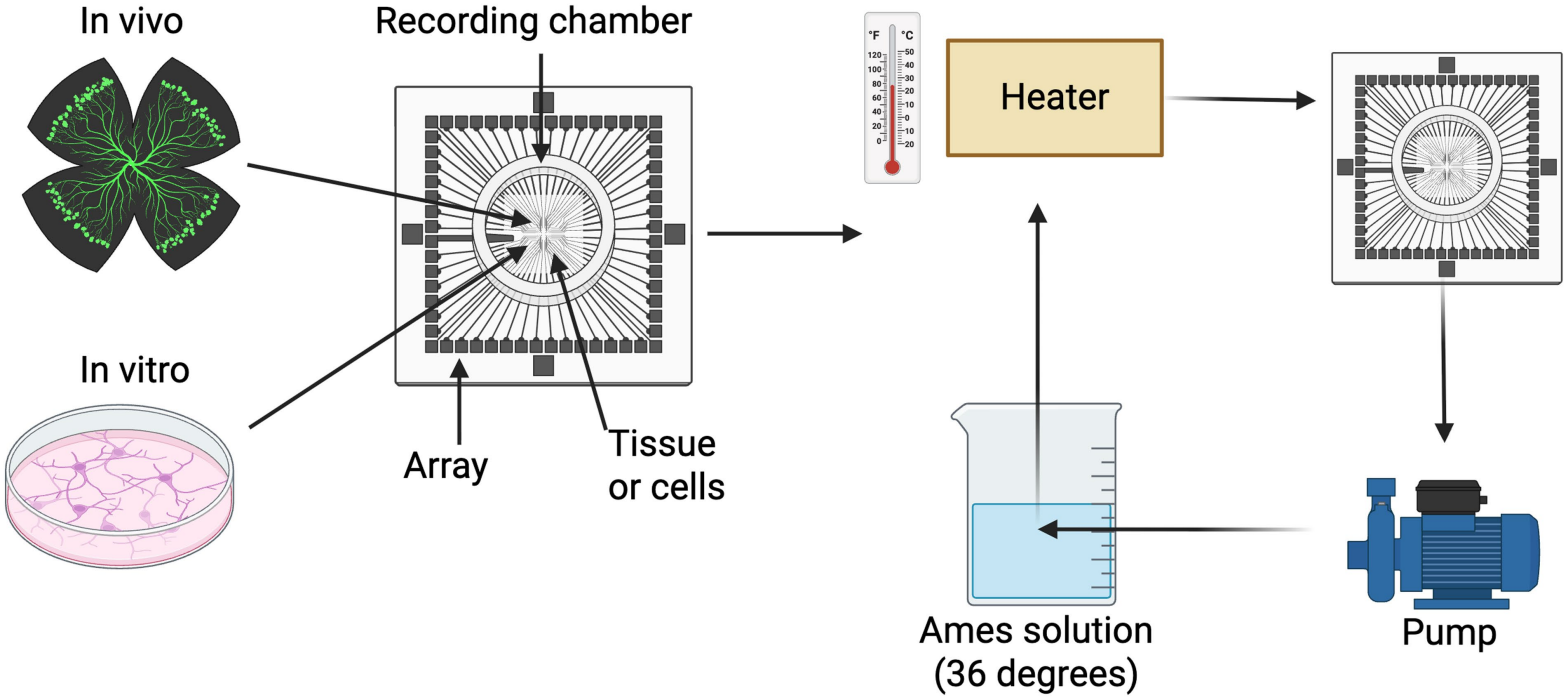
Multielectrode array (MEA) apparatus, setup, and recording. Either cells or tissue are placed on the MEA chamber. A perfusion system is used *in vivo* which is continuously perfused with AMES solution with 95% O_2_ and 5% CO_2_ and well heated to reduce variations in the chamber.

The Multielectrode array (MEA) is a device that consists of multiple microelectrodes for recording or stimulating neurons (Burley and Harvey, 2021). The first implantable array was developed in 1972: a planar MEA with two rows of 15 electrodes, described as recording electrical signals among cells (Thomas et al., 1972). MEA has been adopted to stimulate and record activity along the visual pathway at the levels of the visual cortex, the lateral geniculate nucleus, the optic nerve, or the retina. The setup is illustrated in Figure 4. MEA electrodes transduce currents that depolarise the cell membrane, which in turn opens voltage-gated ion channels and triggers an action potential.

Over time, MEA has been widely used as recording platforms across various models including human retinal explants, non-human primate retina, *rcd1* (progressive retinal atrophy) dog retina, *rd1* mouse retina, and retinal organoids as a functional output for vision restoration. In contrast, MEAs have also been explored as stimulation devices for visual restoration. For example, an 8 × 8 hexagonal MEA array temporarily implanted on the occipital cortex resulted in discrete photic sensation in two volunteers who previously had no light perception, suggesting a role in vision restoration (Dobelle et al., 1974). Very large area electrode arrays for epiretinal stimulation (VLARS) were developed as a flexible MEA consisting of silicon and polyimide and implanted in enucleated eyes of pigs and rabbits using a transscleral approach (Waschkowski et al., 2014). This led to some electrical stimulation and recording of the retina (Waschkowski et al., 2014). However, there were concerns for intra- and pos-operative complications.

In *rd1* mice, treatment with the light-gated excitatory mammalian ion channel called light-gated ionotropic glutamate receptor (LiGluR) targeting retinal ganglion cells, showed light-induced firing recorded by MEA, suggesting restored retinal function in visible light (Gaub et al., 2014). In two mutant *rcd1* dogs who had received an intravitreal injection of AAV2.CAG.LiGluR: expression was observed by confocal microscopy and a restored light response was measured by MEA recording (Gaub et al., 2014). Following this work as a proof of concept, MEA recordings were used to characterise the light responses of retinal ganglion cells in *rd1* mice following AAV2 driving the expression of human rod opsin with meclofenamic acid enhanced effect (Eleftheriou et al., 2017). In non-human primates, wild-type treatment with AAV2.7 m8 ChR-tdT used 256-multielectrode array recordings to show improved spatiotemporal resolution and pattern discrimination, comparable with vision restoration (Gauvain et al., 2021).

Human retinal organoids have been used to assess the efficacy of optogenetic therapies. Human-derived retinal organoids treated with human rhodopsin driven by CAG and packaged into AAV have demonstrated expression dependent on the vector dose and capsid (Shamsnajafabadi et al., 2024). Transplantation of genome-edited retinal organoids has been able to restore some visual function. It was demonstrated at 12 weeks that there was light-evoked and spontaneous activity from retinal ganglion cells recorded by MEA (Watanabe et al., 2025). Another recent application was to transplant genome-edited *Islet1−*/*−* retinal organoid sheets into end-stage *rd1* mice with functional activity measured by MEA to induce ON, OFF and ON–OFF responses to light stimulation (Watanabe et al., 2025). The SpheroGuide MEA system has been developed with an integrated placement funnel to facilitate plating organoids over an electrode. However, there are limitations inherent to the heterogeneity of organoids and spatial resolution due to the number and spacing of electrodes.

The Lumos plate can be paired with the Maestro MEA system to assess real-time functional responses of cells and iPSC-derived retinal organoids (Lumos, 2025). The Lumos and Maestro system has been used to demonstrate functional activation of motor neurons transduced with ChR2 using blue light (Fleming et al., 2025). The advantages of the Lumos system are the optimal delivery and dispersion of light in a multiwell plate, high throughput, and more consistent recordings compared to a multichannel MEA rig. It provides a platform for screening optogenetic therapy that is quicker, with a higher throughput and less complex system setup compared to the multichannel MEA (Lumos, 2025). However, microsystems have less customisation, which would otherwise entail more technical skills, and it may not be as sensitive as a custom-built setup.

MEA assay is an excellent method of evaluating optogenetic tools as potential retinal degeneration therapies. It provides a powerful bridge between single-cell electrophysiology and network-level activity. Unlike patch-clamp techniques, MEAs enable simultaneous recording from multiple neurons and capture spatiotemporal patterns of activity, offering insight into how optogenetic signals propagate across retinal circuits. This output from retinal ganglion cells, such as increased firing rates or pattern discrimination, is directly relevant for visual percepts at higher visual centres, although further downstream processing is to be expected for interpretation of meaningful restored vision. Compared to calcium imaging, MEAs provide superior temporal resolution and direct electrophysiological readouts, but lack cellular specificity and can be limited by electrode density and sampling bias. Furthermore, variability in retinal degeneration models and tissue integrity can influence signal interpretation. As such, MEA is best positioned as a critical intermediate assay for assessing network-level responsiveness and refining candidate optogenetic tools but should be complemented by *in vivo* and behavioural assessments to establish true functional and translational relevance.

## Electrodiagnostic testing

Electrodiagnostic testing plays an important role in testing optogenetic therapy as it allows for the electrical activity to be measured *in vivo* in response to light stimulation. ERG measures retinal function, dorsal lateral geniculate nucleus (dLGN) recordings are the gold standard for assessing retinothalamic transmission, and visual-evoked potentials (VEP) evaluate the functional integrity of the visual pathway from the retina to the visual cortex, including optic nerve function, post-retinal visual pathway conduction and primary visual cortex response to visual stimuli. For retinal input, the largest visual recipient area is the dLGN, which in turn projects primarily to the primary visual cortex (V1) but also to other secondary areas such as the extrastriate cortex and the superior colliculus.

### Electroretinography

ERG provides quantitative assessment of whole-retina function and aims to evaluate optogenetic vision restoration at the retina-level. Opto-mGluR6, an engineered optogenetic actuator targeting ON-bipolar cells, when used to treat *rd1* and *rd10* generated an inverse, electronegative b-wave in the ERG (van Wyk et al., 2015). While ERG remains a non-invasive method for determining the global retinal function, it has a low sensitivity and lacks cellular specificity which it may be difficult to detect functional recovery.

### Dorsal lateral geniculate nucleus recordings

dLGN recordings provide a direct, high-fidelity approach to assessing vision restoration. Light is delivered to the retina, and responses are transmitted via ganglion cell axons to the dLGN neurons, which fire above their baseline activity in response to the light stimulus. These recordings are therefore a direct measure of electrical responses being transmitted from the retina to the brain, closely reflecting retinal ganglion cell input (all or none spikes from individual relay neurons), filtered by thalamic gating. It has been demonstrated that human rod opsin expressed in non-photoreceptor retinal neurons restored vision in blind mice as measured by recordings from the dLGN (Cehajic-Kapetanovic et al., 2015).

### Visual-evoked potential recordings

Visual-evoked field recordings provide an indirect, system-level measure of optogenetic vision restoration in the primary visual cortex (V1). In contrast to dLGN which mainly relays information, V1 integrates information across space and context, and extracts higher order features. V1 responses are therefore often non-linear, more complex and strongly influenced by intracortical circuitry and feedback.

In the context of optogenetic vision restoration, in *rd1* mice whose retinae were treated with an optoswitch LiGluR, the V1 field recordings demonstrated light-induced activity at 400 μ m depth with the highest amplitude, corresponding to cortical layer 4, compared to untreated *rd1 mice* with no responses at all, and wild-type mice with normal large VEP peak amplitudes (Caporale et al., 2011).

dLGN and VEP recordings provide an opportunity to present complex stimuli (e.g., gratings, patterned flashes), to determine contrast sensitivity with spatial tuning, temporal responsiveness, and response to natural stimuli. However, the V1 responses can be limited due to cortical noise and interpretation of signal. Nonetheless, VEP provides a higher-order evaluation of restored visual function resembling real-world conditions.

Collectively, electrodiagnostic approaches provide a hierarchical framework for assessing optogenetic function from retina to cortex, but each modality captures a different level of the visual pathway with distinct limitations. ERG offers a global retinal readout but lacks sensitivity and cellular specificity, making it poorly suited to detect sparse or cell-type–restricted optogenetic activation, particularly in advanced degeneration. In contrast, dLGN recordings, while invasive, provide a more direct and quantitative measure of retinothalamic transmission and are therefore closer to a true physiological correlate of restored signal propagation. However, they remain limited to preclinical models and do not capture perceptual processing. VEP recordings extend this assessment to the cortical level, integrating signals across the visual pathway, but are inherently influenced by cortical processing, feedback, and noise, making interpretation more complex and less specific to retinal input. Importantly, none of these measures directly assess meaningful visual perception, and discrepancies can arise between electrophysiological restoration and behavioural outcomes. Therefore, while electrodiagnostic testing is essential for demonstrating signal transmission and pathway integrity, it should be considered complementary to behavioural and perception-based assays when evaluating the translational potential of optogenetic therapies.

## Behavioural testing

In preclinical studies of vision restoration, behavioural testing is important since it assesses meaningful functional outcomes. While optogenetic therapy may restore cellular function, the light-evoked responses need to be translated into visual perception and processing.

**Figure 5:**
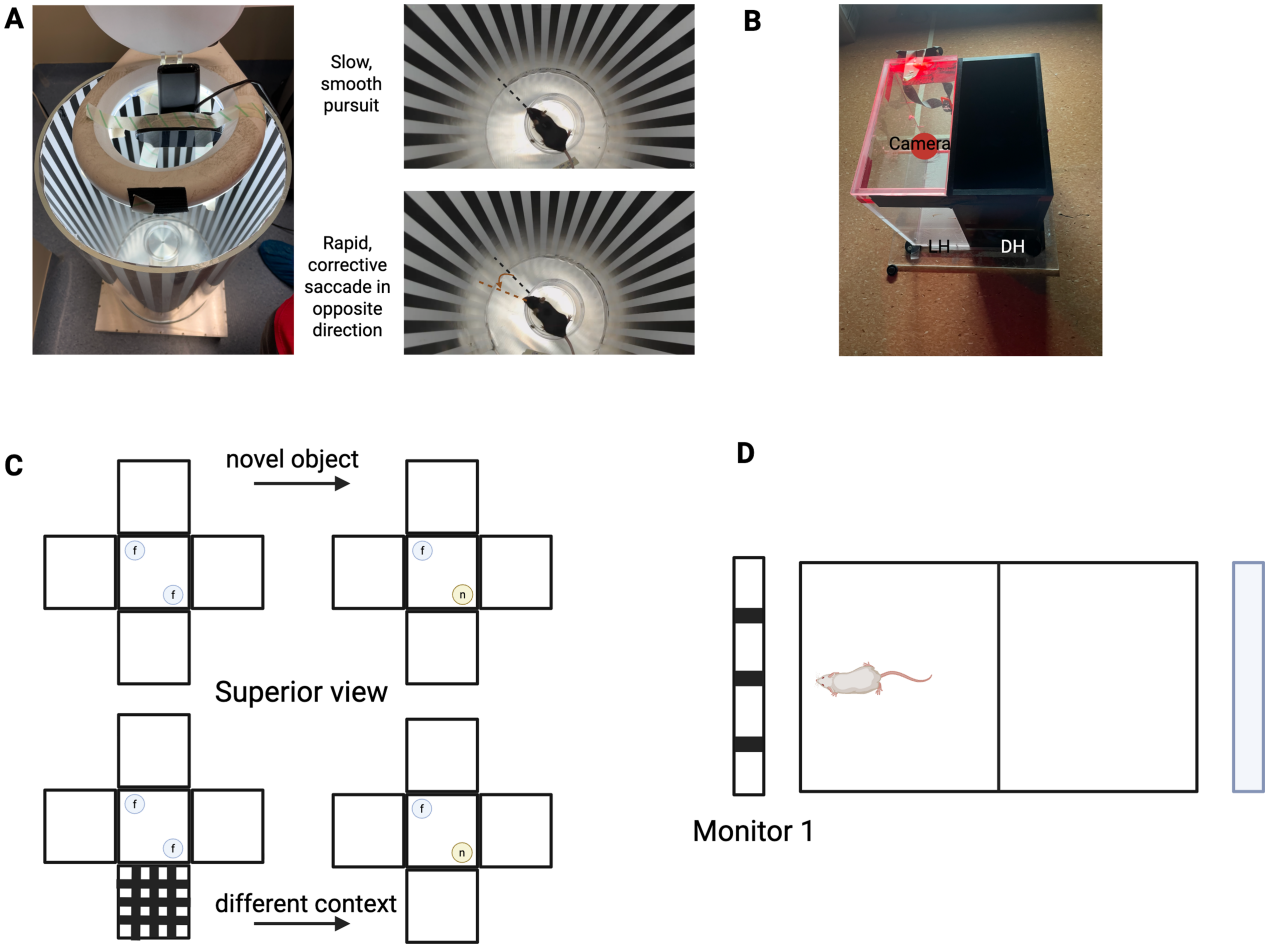
Behavioural functional tests. **(A)** Optomotor response with a rotating drum lined with black and white stripes which can be set at different spatial frequencies. A mouse is placed on a central elevated platform and movements are recorded by a camera. **(B)** The light aversion test involves a dark and light box chamber with a little opening allowing the mouse to navigate between the two. Movements recorded using a camera. **(C)** Object recognition test with a *f* (familiar) and *n* (novel) object introduced and assessment of recognition following a change of context. **(D)** Novel behavioural paradigm showing two monitors at either end of the box, and the behaviour of the mouse is recorded.

There are several types of behavioural testing which include behavioural light–dark avoidance, visual context recognition testing, optomotor response, a novel behavioural test, the visual cliff test, and the water maze test. These are illustrated in Figure 5.

Behavioural light–dark aversion aims to assess light perception and involves a box with equally sized light and dark chambers where animals can freely move between (Takao and Miyakawa, 2006). Each animal is placed in the bright chamber away from the opening to the dark half, lid closed, and movement is measured with a camera in the bright half for 5 min. The time in the light chamber is recorded. If the mouse is able to detect light, then it will prefer the dark component since mice are nocturnal. This provides a simple and rapid assessment indicating basic light perception restored in retinal degeneration following optogenetic therapy and can be used as a screening tool for further complex behavioural assays or electrophysiological testing. Mice treated with ChRmine-T119A packaged into AAV2-4YF showed significant rescue of light-avoidance at ambient light levels at 360 lux (*p* < 0.01) (Fong et al., 2025). However, the assay is not specific to visual acuity or pattern recognition.

The optomotor reflex test measures the detection of moving patterns, and it uses an optokinetic drum that rotates with the mouse in the centre (Prusky et al., 2004). The mouse is placed on a platform and a camera suspended above the drum is used to record eye tracking behaviour following the moving stimulus. It is a reflex movement in response to visual stimuli (rotating stripes), hence an involuntary response which does not require training. It has the advantage of being a non-invasive, quantitative test that can be used to detect if the optogenetic therapy has restored spatial acuity and contrast sensitivity. The test therefore relies on restoration of more complex visual function normally requiring input from horizontal cells for contrast sensitivity and direction selective ganglion cells for horizontal motion.

Visual context recognition testing involves more complex visual processing and depends on the recognition of an object that is context specific (Zhang et al., 2020). Its basis is that an animal that encounters a familiar object in a new environment is less likely to recognise that object. Animals are placed in a box with identical copies of an object in each corner (step 1), with the mouse being able to explore and the movements recorded by a camera. The mouse is then placed in an identical box with a new copy of the familiar object in one corner and a novel object in the other corner (step 2). The outcome measure is the time spent exploring the new object versus the familiar one, and the mouse would be expected to spend more time exploring the novel object. The test is repeated but adding a new visual context in step 1. When the mouse is moved into the second box, it no longer recognises the “familiar” object due to the change in visual context and spends equal amount of time with the familiar and novel objects.

In impaired vison there is change in behaviour between the different scenarios, as the animal has not detected the difference in visual environment. This is relevant since optogenetic therapy aims to improve higher-order vision processing with the integration of visual information and memory. It is therefore used in studies aiming to demonstrate that optogenetic therapy restores form vision, not just light sensitivity. For example, if treated blind mice can successfully recognise a change in visual environment, it suggests that image-forming pathways are at least partially reactivated. Advantages include non-invasive, naturalistic behaviour, which can be repeated over time to assess longitudinal changes in vision. The assay also requires animals to have good cognitive function and mobility. Also, it may not work well in late-stage degeneration without sufficient visual restoration.

The visual cliff test assesses depth perception and is useful for testing binocular vision following optogenetic therapy (Fox, 1965). All species have some capacity for distinguishing depth perception based on visual cues. Rodents are placed on a shallow surface and are observed to determine any sense of reluctance to crawl toward the perceived drop, reflecting depth perception abilities. The advantage of this test is that it evaluates higher-order processing, it is non-invasive and can be quantified.

The visual water maze test is useful in evaluating spatial frequency threshold, contrast sensitivity and visual learning (Frick et al., 2000). This is a hippocampal-dependent test of spatial reference memory where the rodents need to find an escape platform in a tank of water. This is relevant to optogenetic therapy assessment since vision restoration would assist navigation and involve complex visual processing. The visual water maze task is a powerful assay to test visual function, especially in rodents, but it comes with several important disadvantages and limitations. The visual water maze task, while widely used to assess visually guided behaviour in rodents, presents several limitations that complicate its interpretation in vision restoration studies. First, the task imposes high cognitive and motor demands, as it requires learning, memory, and coordinated swimming. This makes it difficult to determine whether poor performance is due to impaired vision, cognitive deficits, or stress-related behaviour. Indeed, swimming is inherently aversive to rodents and can induce significant stress, potentially masking subtle visual improvements and increasing variability in results. The assay is also training-intensive, typically requiring multiple sessions over days, which limits its suitability for fragile animals, such as those with late-stage retinal degeneration. Moreover, it does not specifically quantify visual acuity or contrast sensitivity, as animals may rely on spatial cues or memory to complete the task, thereby compensating for poor vision. In cases of advanced photoreceptor loss, animals may be unable to learn the task altogether, which is particularly problematic for evaluating early-stage optogenetic interventions that may only restore rudimentary light perception. This can lead to variability in results and potentially mask subtle visual improvements. Lastly, environmental variables such as lighting, water temperature, and maze design (some are complex with 6–8 arms) can significantly affect performance, necessitating strict standardisation to ensure reproducibility across studies. Further, the interpretation of behavioural assays requires caution, as residual visually guided behaviour may occur even in the absence of functional rods and cones, as a result of intrinsically photosensitive retinal ganglion cells (ipRGCs) expressing melanopsin. These challenges underscore the importance of using complementary behavioural and physiological measures when evaluating therapeutic efficacy in preclinical models.

Different retinal degeneration models such as *rd1*, *rd10*, and *rho−*/*−* are helpful in assessing optogenetic therapy at different stages of disease, target cells and opportunistic windows for intervention. At times, behavioural testing, however, can involve stressful environments and reflexes rather than goal-oriented behaviour. A simple and less stressful method of assessment can be made using spontaneous behaviour (Cehajic-Kapetanovic et al., 2015). An adaptation to the light–dark box was developed to probe free locomotor behaviour in mice in response to a multitude of light stimuli, including naturalistic scenes. This could be used for longitudinal evaluations of optogenetic treatments. *Rd1* mice treated with grm6-RHO were shown a video of a swooping owl in the behavioural test arena and responded to the stimulus with a significant increase in activity (Cehajic-Kapetanovic et al., 2015). This response was also seen in wild-type mice but not in control mice.

Behavioural assays represent the most relevant surrogate for meaningful visual function in preclinical models and therefore hold a critical position in the translational pipeline for optogenetic therapies. Unlike molecular or electrophysiological measures, these tests assess the integration of visual signals into perception-driven behaviour, which is the end goal of vision restoration. That said, their interpretation is inherently complex, as performance is influenced by multiple non-visual factors including cognition, motivation, stress, and motor function. This is particularly problematic in advanced retinal degeneration models, where animals may fail tasks despite partial restoration of visual function. Moreover, many commonly used assays, such as light–dark avoidance or optomotor response, predominantly assess lower-order visual capabilities (e.g., detection or reflexive tracking) and may overestimate functional recovery without demonstrating true image-forming vision. In contrast, tasks assessing object recognition, context discrimination, or naturalistic behaviour offer a closer approximation to higher-order visual processing but are less standardised and more variable. Therefore, behavioural testing should be carefully aligned with the expected level of visual restoration and interpreted alongside electrophysiological data. Critically, this highlights the need for prioritising recognition-based and perception-driven endpoints, which are more likely to reflect clinically meaningful outcomes and better inform the design of human trials.

## Optogenetic therapy in clinical trials

Preclinical work of optogenetic therapy establishes a foundation for meaningful clinical translation. The level of efficacy demonstrated in preclinical studies for each opsin undergoing testing in current clinical trials is summarised in Table 1. Across these studies, there is substantial heterogeneity in trial design, including differences in vector platforms, target cell populations, delivery routes, and critically, functional endpoints used to assess efficacy. This variability makes direct comparison between studies challenging and highlights the absence of a standardised framework for evaluating clinical success in optogenetic therapy.

**Table 1:** Preclinical and clinical trial outcomes for opsins in current optogenetic therapy clinical trials to treat retinal degeneration.

Company	Phase	Opsin	Vector	NCT	Preclinical functional tests	Vision function at recruitment	Primary outcome	Secondary outcomes
AbbVie	I/IIa	ChR2	rAAV2	02556736	Patch clamp, MEA and VEP	Worse than HM or CF to 20/200	Safety and tolerability.	Not reported.
GenSight Biologics	I/IIa	ChrimsonR	rAAV2.7 m8	03326336	Patch clamp, MEA	Worse than CF	Safety and tolerability.	BCVA, VF, mobility, quality of life, immune response
Bionic Sight	I/IIa	ChronosFP	rAAV2	04278131	Patch clamp, MEA	LP in at least one eye	Safety and tolerability.	Light detection, shape and motion detection by Diagnosys visual function testing
Nanoscope Therapeutics	I/IIa	MCO1	rAAV2	04919473 05417126	Behavioural testing with OKN, water maze	LP or NLP in the study eye	Safety and tolerability.	BCVA, light-guided mobility, determination of shape, determination of optical flow.
Nanoscope Therapeutics	IIb	MCO-010	rAAV2	04945772	VEP, behavioural testing with OKN and water maze	1.95 to 2.25 LogMAR	Change in vision-guided mobility	BCVA, Multi-luminance Y-mobility Test
Zhongmou Therapeutics	I	PsCatCh2.0	AAV2	06292650	VEP, patch clamp, behavioural testing	Worse than CF	Safety and changes in IOP	Full stimulus test, multi-luminance mobility test, quality of life, BCVA, VF
Ray Therapeutics	I	Proprietary opsin	AAV7m8	06460844	Not publicly available	Not specified.	Safety and tolerability.	BCVA, LLVA, multi luminance mobility, contrast sensitivity, full-field static VF, low vision quality of life

Clinical trial outcomes are outlined on ClinicalTrials.gov. BCVA = best corrected visual acuity, low luminance visual acuity = LLVA, VF = visual fields, LP = light perception, HM = hand movements, CF = count fingers, MEA = multielectrode array, VEP = visual evoked potential, OKN = optokinetic nystagmus.

For example, ChR2 delivered by AAV demonstrated long-term expression and restored vision measured by whole-cell patch clamp, MEA and VEP in *rd1* mice. This foundation led to AbbVie (previously Allergan) with the intravitreal delivery of ChR2. Meanwhile, the GenSight PIONEER trial using ChrimsonR opsin delivered in the AAV 7m8 vector was effective as measured by several functional assays (Douar et al., 2016). Electrophysiological recordings by MEA and patch clamp along with expression were assessed up to 6 months (Douar et al., 2016). MEA demonstrated light responses in all retina with 3 of 4 retina having high amplitudes in response to light as high as 360 Hz. In patch clamp experiments, there were large photocurrents recorded in 3 of 4 retinas (Douar et al., 2016). The efficacy of the AAV2 vector and Chronos as the optogenetic protein was demonstrated by the production of robust electroretinograms in *rd1* in a dose-dependent manner (Yan et al., 2023). Notably, while these preclinical studies demonstrate robust electrophysiological and cellular responses, the extent to which these outputs predict clinically meaningful visual improvement remains uncertain, especially given the need for supraphysiological light activation levels in artificial settings. This reflects a broader challenge in the field, where strong preclinical functional signals do not always translate into measurable patient benefit.

Meanwhile, MCO1 continued into clinical trial following expression in *rd10* mice and behavioural testing with the optokinetic response and water maze (Batabyal et al., 2021). There was no published characterisation of the kinetics or temporal resolution in the preclinical stage. The phase I/IIa study assessed visual acuity, visual field, Y-mobility test, A-mobility test and low vision multi-parameter test in four patients (Mohanty et al., 2025). It is also important to note the eligibility criteria specified a visual acuity of worse than 1.9 logMAR in the study eye and no better than 1.6 logMAR in the fellow eye at screening, i.e., the fellow non-study eyes had good vision, suppressing the image in the poorer eye. It cannot be excluded that given the asymmetric vision, an improvement in the poorer eye, through training and improved fixation, may also improve the vision in the study eye. The study results therefore need to be interpreted with caution. The Freiburg visual acuity test showed improvement in four patients, however the best corrected visual acuity (BCVA) was compromised by visual opacities. The Y- and A-mobility tests demonstrated decreased latency and improved mobility test scores following treatment. While mobility tests have their limitations in poor standardisation, learning effects and subjectivity, they provide some level of proof of concept. At 52 weeks, the low-vision multi-parameter test demonstrated a decrease in the mean value of direction accuracy and speed threshold which was attributed to be due to a loss of participation of patient 2 and poor performance of patient 4, who developed vitreous haze. This example underscores the importance of rigorous preclinical characterisation, particularly with respect to MCO’s cellular expression profile, temporal resolution and signalling kinetics, which are likely to influence the quality of restored vision. This inconsistent depth of preclinical evaluation across programs further complicates interpretation of clinical outcomes.

Functional assays in preclinical studies play an important role in forming a foundation and guiding the design of clinical trial primary and secondary outcomes measured to assess vision restoration. There is a need for preclinical behavioural testing and electrophysiology tests to demonstrate higher-order visual function. There are several parallels between preclinical and clinical testing, including the need to assess safety, durability and the level of efficacy in restoring vision to progress forward. The behavioural tests for mice could provide preclinical predictions for assessing functional vision gain with real-world relevance in humans. Since the majority of clinical trials in retinal optogenetic therapy are early-phase, the primary endpoints are safety and tolerability, with one phase IIb trial where the primary endpoint is change in vision-guided mobility (Table 1). However, there is currently no consensus on which clinical endpoints best capture meaningful visual restoration in this patient population. The use of diverse and sometimes non-comparable endpoints across trials further limits the ability to benchmark efficacy and identify best-performing approaches.

As in preclinical studies, concerns about melanopsin-dependent visual learning may also apply to mobility-based functional endpoints in human trials of optogenetic vision restoration. Notably, the multi-luminance mobility test (MLMT) or light-guided navigation tasks, used in trials such as those by GenSight (GS030, secondary endpoint) and Nanoscope (MCO-010, primary and secondary endpoints), assess patients’ ability to navigate a maze or avoid obstacles under varying light intensities. While improvements in mobility scores are encouraging, it remains unclear whether these reflect meaningful image-forming vision or are driven by brightness discrimination via melanopsin-expressing ipRGCs, which are preserved in late-stage retinal degeneration. Given that melanopsin-driven responses are slow and coarse but sufficient for learning and adaptation over repeated testing, training effects or compensatory brightness-based cues could allow patients to improve performance without restoration of detailed visual processing. This raises the need for more stringent endpoints in future trials, including forced-choice object recognition, motion detection, or contrast sensitivity testing, ideally complemented by neuroimaging or non-invasive electrophysiological recordings to corroborate cortical visual processing (Hillier et al., 2017). Differentiating between true spatial vision and luminance-guided navigation will be essential to validate the therapeutic value of optogenetic interventions. This challenge is compounded by the potential for behavioural adaptation over time, where repeated exposure to testing paradigms may lead to improved performance independent of true visual restoration. As such, distinguishing between genuine perception-driven improvements and learned or compensatory behaviours remains a critical unmet need in clinical trial design.

Taken together, current clinical trials can be broadly categorised based on their primary functional readouts: electrophysiological-driven validation, mobility-based endpoints, and visual acuity–based measures. Each approach captures different aspects of visual function, but none alone fully reflects meaningful visual perception, reinforcing the need for multimodal and standardised outcome measures.

RTx-015, a proprietary opsin, has been developed to target retinal ganglion cells using the AAV7m8 vector. A Phase I, non-randomised dose-escalation study is recruiting retinitis pigmentosa patients to receive treatment at three doses. Also targeting retinal ganglion cells, a novel engineered ChR variant opsin, PsCatCh2.0, has been developed that has a light-sensitivity which is 100-fold lower than the previous. Zhongmou Therapeutics is now active but not recruiting for the phase I escalating trial in patients with retinitis pigmentosa. It has been reported that 66.7% of patients showed clinically significant improvement in best corrected visual acuity (0.42 logMAR) and 88.3% had an improvement in at least two of three multi-luminance tests (Zhou et al., 2025).

A single-armed investigator-initiated trial was performed to assess the safety and efficacy of UGX-201, a modified opsin fusion protein, in advanced retinitis pigmentosa patients (CHICTR identifier: ChiCTR2200062174). There were two cohorts: cohort 1 (*n* = 6 with light perception and BCVA of the worse eye <0.02) and cohort 2 (*n* = 3 lacking light perception). It was reported to be safe and the cohort 1 vision had a mean change of −0.28 logMAR from baseline at 52 weeks follow up, while two of three participants regained light perception at the most recent 24 week visit (Luo et al., 2025).

Overall, these findings highlight a central challenge in the clinical translation of optogenetic therapies: the disconnect between measurable biological activity and meaningful visual outcomes. Addressing this will require greater alignment between preclinical functional assays and clinical endpoints, alongside the development of standardised, perception-driven measures of vision restoration.

## Critical discussion of functional methods for vision restoration

**Figure 6:**
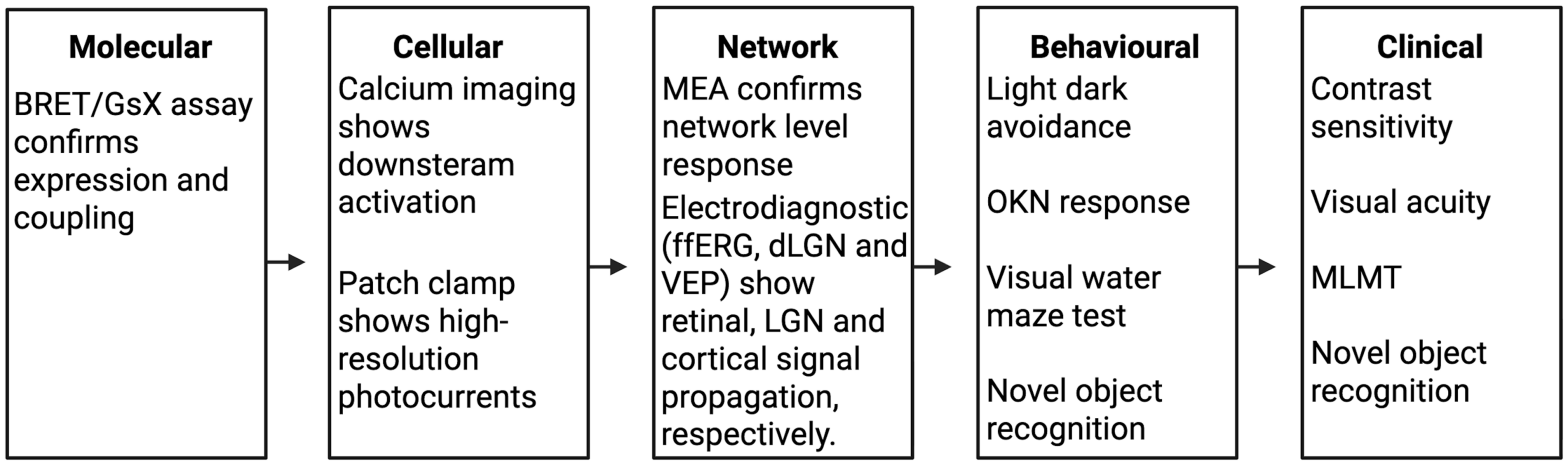
Translational framework for functional methods. Step-wise approach including molecular, cellular, network, behavioural, and clinical functional evaluation of optogenetic therapy. BRET = bioluminescence resonance energy transfer, GsX = G protein signalling, MEA = multielectrode array, ffERG = full field electroretinography, dLGN = dorsal lateral geniculate nucleus, VEP = visual evoked potential, OKN = optokinetic nystagmus, MLMT = multi-luminance mobility test.

The most appropriate functional assay depends on the disease model, optogenetic properties, and the aspect of visual function to be assessed (Table 2). To assess the effectiveness of optogenetic therapy, there are considerable concerns regarding the reproducibility given variability in light intensity, stimulation protocols and model systems, along with a lack of standardisation or framework to guide functional assessment which are essential for therapeutic success. This variability contributes to a lack of reproducibility across studies and highlights the need for standardised functional assessment frameworks. A translational framework has been proposed to guide how each functional method could be integrated in a coherent pipeline (Figure 6). Critically, these approaches can be conceptualised as a hierarchical and sequential pipeline, progressing from molecular validation to behavioural and perceptual outcomes. Each level provides distinct and complementary information, with increasing biological and translational relevance, but decreasing experimental control and throughput. This framework enables rational selection and prioritisation of assays based on the specific stage of therapeutic development.

**Table 2:** Comparison of functional assays based on input, method, output, advantages and disadvantages.

Considerations	BRET/GsX assays	Calcium imaging	Patch clamp	MEA	Electrodiagnostic tests eg ERG, dLGN recordings, and VEP
Model system	Pair of proteins – labelled either with a bioluminescent donor or fluorescent acceptor	Fluorescent calcium-sensitive dyes or genetically encoded calcium indicators	Cells, tissues or membrane patches with an electrolyte solution	Neuronal cells are grown on a grid of microelectrodes.	*In vivo*
Method	BRET relies on energy transfer from a bioluminescent donor molecule to a fluorescent acceptor molecule when nearby.	Cells or tissues are loaded with a calcium-sensitive dye which fluoresces in response to binding calcium.	Cell-attached, whole-cell, inside out and outside out recording modes	Cells or tissue are placed onto electrodes that record extracellular electrical signals.	ffERG measures global retinal electrical response by electrodes Light evoked spikes in activity from neurons to dLGN VEP measures cortical response to stimuli by electrodes
Output	Protein–protein energy transferred	Changes in intracellular calcium	Ionic currents recorded based on response to stimuli	Extracellular electrical activity	ffERG: A-wave, B-wave amplitudes and implicit times dLGN: spikes in activity VEP: P100 latency and amplitude
Advantages	Live cell real-time Quantitative High temporal resolution	Spatial resolution Non-invasive Real-time	High temporal and spatial resolution Direct measurement	Non-invasive Network electrical activity Scalability	Objective ffERG: global retinal function dLGN: retinal activity to the brain VEP: optic nerve or visual pathway conduction
Disadvantages	Limited to close interactions within a distance 1 – 10 nm Requires genetic engineering Sensitivity to expression levels	Indirect measurement Photobleaching and phototoxicity Temporally calcium is slower In wholemounts, the thickness can cause light scattering and excitation	Technically challenging Invasive Set up is complicated Single cell measurements	Lower temporal resolution then patch clamp Spatial resolution limited by electrode spacing Expensive, specialised equipment and expertise	Limited spatial resolution (global not focal) VEP can be affected by attention and refraction Requires specialised equipment
Example in optogenetics	Modulation of temporal resolution measured by BRET assay (Rodgers et al., 2023)	Channel rhodopsin for photocontrol of calcium signalling (Fernandez Lahore et al., 2022)	Melanopsin-mGluR6 chimera targeting bipolar cells restores vision (Kralik et al., 2022)	Light-gated ionotropic glutamate receptor (LiGluR) targeting retinal ganglion cells showed light-induced firing recorded by MEA (Gaub et al., 2014)	ERG of *rd1* mice treated with Opto-mGluR6 demonstrated an inverse, electronegative b-wave (van Wyk et al., 2015). Electrophysiological responses to light were evoked in the dLGN following treatment of grm6-RHO in *rd1* mice (Cehajic-Kapetanovic et al., 2015). LiGluR treated *rd1* mice showed restoration of VEP in the primary visual cortex (Caporale et al., 2011).

A key limitation across functional assays in optogenetic research is the lack of standardisation, which poses significant challenges for reproducibility and cross-study comparison. Functional outcomes are highly sensitive to experimental variables, including light intensity, wavelength, stimulation paradigms (e.g., pulse duration, frequency), expression levels of optogenetic constructs, and the choice of disease model. For example, differences in illumination conditions alone can significantly alter apparent efficacy, particularly for opsins with varying light sensitivities. Similarly, variability in retinal degeneration models, vector delivery methods, and timing of intervention can influence both the magnitude and interpretation of functional responses. At the assay level, discrepancies in electrode density in MEA recordings, indicator selection in calcium imaging, or behavioural task design further complicate direct comparisons between studies. The absence of standardised protocols or reporting frameworks limits the ability to benchmark optogenetic tools and identify best-in-class candidates. Therefore, there is a clear need for harmonised experimental guidelines, including standardised stimulation parameters, reporting of light exposure conditions, and adoption of comparable functional endpoints. Such standardisation will be essential to improve reproducibility, enable meaningful comparison across studies, and facilitate translation of optogenetic therapies into clinical practice.

A combination of functional assays provides justification for translating optogenetic therapy into a clinical application. BRET/GsX assay guides opsin suitability at a molecular level and is focused on G protein coupling for activation. Meanwhile, calcium imaging subsequently evaluates on a cellular level whether the opsin delivered can lead to light-evoked responses. The highest measure of spatial and temporal resolution is patch clamp on a cellular level, which is the gold standard method for direct membrane potential, although it is for single-cell analysis and involves a technical setup. Calcium imaging provides high spatial resolution but not temporal resolution, and can be used to measure across large networks of neurons. Thus, calcium imaging represents a versatile, scalable, and informative platform for early-phase screening in the development of effective optogenetic therapies for vision restoration. MEA provides a network level analysis of activity with high temporal and spatial resolution based on electrode density. Behavioural activity, as measured by optomotor response test, maze navigation or light avoidance, is helpful in assessing different aspects of visual function. In practice, this pipeline begins with high-throughput molecular and cellular assays (e.g., BRET, calcium imaging) to establish opsin functionality and signalling properties, followed by electrophysiological validation (patch clamp, MEA) to confirm neuronal activation and network integration. This is subsequently extended to *in vivo* pathway-level assessments (ERG, dLGN, VEP) to demonstrate signal propagation, before ultimately evaluating behavioural outputs that approximate visual perception. Progression through these stages is not merely additive but serves as a series of translational filters, where failure at any level may indicate limited clinical potential.

Advances in optogenetic therapies are focused toward improving vector safety, sensitivity, kinetics, and capsids/promoters, so functional assays need to detect, quantify and provide critical insights for further development. The selection of the most appropriate objective functional assay for testing each opsin depends on the properties, kinetics and spatiotemporal resolution. For example, Chronos has fast kinetics, which may be best measured by patch clamp due to its high temporal resolution, which is important for detection (Klapoetke et al., 2014). Meanwhile, calcium imaging may better measure opsins with longer deactivation times, such as melanopsin (Renteria et al., 2025). An appropriate functional assay also depends on the target and level of neural activity, which can be further evaluated by electrophysiology to assess neural stimulation of the LGN and cortex. This is relevant considering the retinal remodeling which can affect rewiring and changes in synapses which can change the signalling pathways (Jones et al., 2003; Marc et al., 2003). Further electrophysiological testing could be helpful to determine what extent of retinal architectural changes would be acceptable and suitable for optogenetic therapy. Given these examples, not all assays carry equal weight in predicting translational success. Early-stage assays are optimised for sensitivity and throughput but may overestimate efficacy, whereas later-stage assays provide greater physiological relevance but are more variable and resource-intensive. Therefore, prioritisation should favour assays that align most closely with the intended mechanism of action and expected clinical outcome, rather than relying on any single modality.

The delivery of an opsin may not be sufficient if the downstream cascade components (e.g., transducin, c-GMP, etc.) are not present. Functional assays such as BRET assay quantify and detect coupling but this may not be helpful in evaluating function if the visual cycle components are lacking downstream. To address this, quantitative proteomics and single-cell transcriptomics could be useful in advanced retinal disease to determine what components are present, in different potential target cells and at what levels before continuing into clinical trials. There may be need for functional assays that not only focus on G-protein coupling or calcium channel signalling but also detect activation of specific downstream elements. This highlights a critical translational gap in the field: strong molecular or cellular activation does not necessarily predict restoration of meaningful vision. Bridging this gap requires integration of functional assays with complementary approaches that assess circuit integrity and downstream processing, rather than relying solely on proximal signalling readouts.

Human opsins are considered preferable and advantageous with decreased vector-related intraocular inflammation, enhanced light sensitivity and reduced phototoxicity risks (Bica and Cehajic-Kapetanovic, 2025). However, there needs to be consideration given to chromophore recovery. For instance, human opsins bind 11-cis retinal to be photosensitive however this may not be available in advanced degeneration. Systemic 9-cis retinal supplementation, bistable pigment chimeras and co-expression of retinal isomerases have demonstrated short-term restoration in mice. However, longer-term and larger scale need to be performed. A functional assay that could be useful is quantitative analysis of chromophore turnover in human tissue.

Each functional assay needs to be carefully interpreted in the context of the retinal degeneration model used considering the strengths and weaknesses of each model. Retinal organoids provide a cellular model however do not fully replicate the architecture, vasculature, cellular expression and differentiation or circuit connectivity which limits their utility for evaluating more complicated network integration. Meanwhile, there is no ideal animal models for assessing the translational potential of optogenetic therapy and each model is limited in its ability to recapitulate the human eye. The *rd1* mice have been commonly used to evaluate optogenetic therapy since there is a rapid loss of photoreceptors although there is clinical heterogeneity in retinal degeneration phenotypes. In comparison, *rd10* mice have a slower rate of retinal degeneration which may be better at determining the effect of treatment at different stages of degeneration. Rodent eyes are much smaller compared to human eyes, there is a relatively larger surface area for transduction which may overestimate the transduction and there is a higher risk for intraoperative complications including lenticular damage given the large lens or risk for vitreous haemorrhage or detachment. Further, ILM is much thinner in mice which may be more permissive to the virus (Cehajic-Kapetanovic et al., 2011, 2018). As a result, efficacy observed in preclinical models may not directly translate to human disease, reinforcing the need for assay strategies that are robust across models and predictive of clinical outcomes.

Interpretation of behavioural assays in preclinical models of retinal degeneration requires caution, as residual visually guided behaviour may occur even in the absence of functional rods and cones, due to the activity of intrinsically photosensitive retinal ganglion cells (ipRGCs) expressing melanopsin. Several studies have demonstrated that mice with severe retinal degeneration (e.g., rd/rd cl) retain the ability to perform visually guided tasks such as Morris water maze navigation, light avoidance, and object exploration under bright illumination, despite lacking classical photoreceptors (Mrosovsky, 1994; Hatori et al., 2008). These behaviours are abolished in triple-knockout mice lacking melanopsin (Opn4−/−), confirming that they are mediated by ipRGCs. This highlights a major caveat in interpreting results from optogenetic or cell-based therapies using behavioural endpoints alone: apparent functional vision may arise from residual melanopsin-driven light perception, rather than true restoration of spatial or pattern vision. Therefore, the use of melanopsin-deficient animal models, in combination with objective measures such as visually evoked potentials or two-photon calcium imaging, is essential to validate that restored behaviour reflects image-forming vision rather than non-specific luminance detection. This reinforces the necessity of combining behavioural assays with objective electrophysiological and imaging measures to ensure that observed responses reflect true image-forming vision rather than non-specific luminance detection. Such multimodal validation is essential for translating preclinical findings into clinically meaningful outcomes.

In the clinical setting, endpoints have been focused on achieving clinically meaningful benefit. The endpoints in optogenetic therapy have included visual acuity, full-field stimulus threshold (FST) for light sensitivity, contrast sensitivity, multi-luminance mobility test (MLMT) and novel object recognition (Thirunavukarasu et al., 2025; Buckley et al., 2021). Visual acuity is the most common clinical endpoint however it is not suitable for patients with phenotypes amenable to optogenetic therapy who would likely have foveal involving disease with poor visual acuity at baseline. In low-vision settings, the floor effect can confound the quantification of deterioration or improvement. FST challenges patients using pulses of light with different intensities and has been considered practical and of good clinical value. MLMT involves navigating an obstacle course at several level levels which was the primary endpoint in the trial for Luxturna, gene therapy with regulatory approval. Although this is time intensive, requires significant physical space and it is unclear how this may be monitored outside of clinical trial conditions. It also remains unclear how each of the clinical endpoints correspond to the level of expression of optogenetic therapy either *in vitro* or *in vivo*, and pre-clinical method functional outcomes. However, what is important is that true visual restoration is distinguished from compensatory strategies including reliance on residual perception or non-visual cues so there is a need for multiple functional methods for validation.

Together these considerations emphasise that no single functional assay is sufficient to define therapeutic success. Instead, a structured, multi-level framework which integrates molecular, cellular, network, and behavioural assessments, is required to bridge the gap between mechanistic activity and meaningful visual restoration. Alignment of preclinical endpoints with clinically relevant functional outcomes, particularly those reflecting perception and recognition, will be essential for the successful design of phase 3 trials and regulatory approval of optogenetic therapies.

## Conclusion

In conclusion, the functional assay that is used to assess restoration of visual responses needs to be context-specific to the aspect of optogenetics which is being optimised. Each assay provides a better understanding of optogenetic therapies by evaluating both quantitative and qualitative functional measures of preclinical therapeutic effectiveness. There are several different methods of functional testing in cells which each have their advantages, disadvantages and limitations. For instance, patch clamp provides a direct measurement of membrane potential with high spatial and temporal resolution, whereas calcium imaging can give an indirect measure of neuronal activity via cellular calcium influx, and MEA spatial resolution is limited by electrode distribution. Electrophysiology testing *in vivo* provides insightful quantifiable output to measure vision restoration following optogenetic treatment; however, it requires a more technical setup compared to *in vivo* behavioural testing. Behavioural testing provides valuable translatable information for how optogenetic therapies can improve and restore vision. As more optogenetic clinical trials emerge, it will be helpful in determining how each functional measure may correlate with vision gain. A multifaceted approach to the functional preclinical assessment of vision restoration is important to evaluate the efficacy of optogenetic therapy moving forwards into the clinic (Borchert et al., 2024; Ng et al., 2025).

The key message here is that these functional assays should not be considered in isolation but rather integrated within a hierarchical translational framework, progressing from molecular and cellular validation to network, pathway, and behavioural assessment. This is particularly critical given that early-stage functional success does not necessarily predict meaningful visual restoration. Therefore, aligning preclinical functional outputs with clinically relevant endpoints, particularly those reflecting perception and recognition, will be essential to guide the design of future clinical trials and maximise the likelihood of successful translation.
